# Cellulose Ether/Citric Acid Systems Loaded with SrTiO_3_ Nanoparticles with Solvent-Tailored Features for Energy-Related Technologies

**DOI:** 10.3390/molecules30153271

**Published:** 2025-08-05

**Authors:** Raluca Marinica Albu, Mihaela Iuliana Avadanei, Lavinia Petronela Curecheriu, Gabriela Turcanu, Iuliana Stoica, Marius Soroceanu, Daniela Rusu, Cristian-Dragos Varganici, Victor Cojocaru, Andreea Irina Barzic

**Affiliations:** 1 “Petru Poni” Institute of Macromolecular Chemistry, 41a Grigore Ghica Voda Alley, 700487 Iasi, Romania; albu.raluca@icmpp.ro (R.M.A.); mavadanei@icmpp.ro (M.I.A.); stoica_iuliana@icmpp.ro (I.S.); soroceanu.marius@icmpp.ro (M.S.); rusu.daniela@icmpp.ro (D.R.); varganici.cristian@icmpp.ro (C.-D.V.); 2 Faculty of Physics, “Alexandru Ioan Cuza” University, Blv. Carol I, no.11, 700506 Iasi, Romania; gabriela.irina@yahoo.com; 3 “D. Ghiţu” Institute of Electronic Engineering and Nanotechnologies, Technical University of Moldova, Academy Street 3/3, 2004 Chisinau, Moldova; victor.cojocaru@iien.utm.md

**Keywords:** cellulose ether, ceramic nanoparticles, solvent effect, infrared spectroscopy, morphology, colorimetry, refractometry, dielectric behavior

## Abstract

This work aimed to advance the knowledge in the field of eco-friendly dielectrics with applicative relevance for future energy-related technologies. New multicomponent composites were prepared by using a cellulose ether/citric acid mixture as the matrix, which was gradually filled with strontium titanate nanoparticles (5–20 wt%). In this case, citric acid can act as a crosslinking agent for the polymer but also can react differently with the other counterparts from the composite as a function of the solvent used (H_2_O and H_2_O_2_). This led to considerable differences in the morphological, thermal, optical, and electrical characteristics due to distinct solvent-driven interactions, as revealed by the infrared spectroscopy investigation. Hence, in contrast to H_2_O, the oxidizing activity of H_2_O_2_ led to changes in the surface morphology, a greater transparency, a greater yellowness, an enhanced refractive index, and higher permittivity. These data provide new pathways to advance the optical and dielectric behavior of eco-compatible materials for energy devices by the careful selection of the composite’s components and the modulation of the molecular interactions via solvent features.

## 1. Introduction

Science has advanced considerably in many areas, producing novel findings, improved analyses, and a broader knowledge. This progress is fueled by a continuous search for information, the formulation of new materials, characterization techniques, and the elucidation of previously undiscovered phenomena. The development of advanced materials does not only rely on the original association of compounds but also resides in the refinement of molecular interactions in the designed systems [1,2]. For instance, when making polymer-based components for a targeted technology, they are processed into solid layers from a solution phase; hence, the nature of the intermolecular solvent/macromolecule forces determines conformational changes at a molecular level and leads to different properties [3,4,5]. In the past decades, polymer properties were additionally tuned by the reinforcement with numerous fillers/additives to match the criteria demanded by practical uses in domains such as food packaging [6], flexible electronics [7], and energy production [8,9] or storage [10,11]. Currently, the majority of energy-related devices, like solar cells (SCs) and electrostatic capacitors (ECs), require the fabrication of components based on polymer dielectrics.

For SCs in the superstrate configuration, the radiations first reach the glass substrate before they could be absorbed by the layers from the SC active zone [12]. However, the cover glass has some drawbacks, such as a high weight, rigidity, and a low refractive index. These aspects are limiting for the possibility of mounting the SC on curved installations, while limiting light transmission due to reflection losses [13]. The aforementioned technical issues could be addressed by replacing the glass sheet with polymers that display a good combination of characteristics: lightweight, flexibility, thermal resistance, and optical properties [14]. Concerning the latter, in previous reports [8,15,16] we demonstrated that a proper balance between the transparency and refractive index of the polymer cover is essential for minimizing the optical losses in SCs.

In the case of ECs, polymer–ceramic composites have attracted a great deal of attention as reflected in the high number of reports dealing with this topic [17,18,19,20]. In the context of plastic pollution and electronic waste that harmfully affects the environment [21,22], new trends in this domain are shifted more and more towards the utilization of biodegradable and/or low toxicity materials and the elaboration of ‘green’ techniques for composite preparation [23,24]. As a result, natural polymers (e.g., cellulose, chitin, starch, polypeptides, etc.) and biodegradable synthetic polymers (e.g., polylactic acid, polyvinyl alcohol, polycaprolactone) are currently explored for manufacturing devices for energy storage [24]. For designing eco-friendly dielectric composites, lead-free ceramic fillers (e.g., bismuth ferrite, zinc oxide, bismuth titanate, potassium sodium niobate, lithium niobate, aluminum nitride, barium titanate, barium calcium titanate zirconate, strontium titanate (SrTiO_3_—denoted here as ST)) are preferred since lead is found to produce devastating effects on the health of living organisms, destroying eco-systems [25].

Among the lead-free fillers, ST is an invaluable compound in modern technologies, offering a good balance between the electrical features (permittivity, ferroelectricity), thermal stability, resistance to photocorrosion, and tunable microwave behavior [26]. This ceramic material is often prepared by the solid-state reaction of SrCO_3_ or SrO with TiO_2_, at highly elevated temperatures or by hydrothermal procedures; thus, small amounts of SrCO_3_ concomitantly appear during the ST synthesis [27,28]. The investigations on ST-filled polymers for energy-saving purposes are focused on using only synthetic polymers as a matrix, such as polycarbonate [29], polyetherimide [30], and polyvinylidene [31]. According to our knowledge, there is no study on biodegradable polymers and ST for energy-saving applications.

In regard to eco-compatible polymers, cellulose is by far the most abundant in our world, particularly being found in plants. However, this biopolymer displays some processing drawbacks related to its insolubility in water and other solvents [32]. This led to the chemical modification of cellulose, rendering numerous structures of derivatives [33]. Hydroxyethylcellulose (HEC) is a hydroxyethyl ether of cellulose, which has the major advantages of water solubility, excellent thickening, adhesion, optical clarity, and good film-forming properties [34,35,36]. In the presence of citric acid (CA), HEC can be crosslinked, and this enhances some properties, like mechanical strength and stability [37]. The electrical properties of HEC have been addressed by some reports [38,39,40,41]. Liedermann et al. [38] investigated the dielectric relaxation in HEC films, revealing a pronounced relaxation process in the temperature interval of 150–250 K. Ulutas et al. [39] studied the role of the HEC layer thickness on the magnitude of permittivity. Other articles [40,41] examined the effect of additives/fillers on the dielectric behavior for various uses. As far as we know, no paper on HEC/ST composites for energy saving has been published, and the only studies on HEC’s involvement in the preparation of dielectrics for energy saving are represented by our previous works, where this biopolymer was loaded with bentonite clay [42], walnut leaves [43], or nettle leaf ash [44].

The current article is a continuation of our previous efforts on making environmentally friendly dielectrics for energy-related devices. Here, novel multicomponent dielectric materials were prepared by combining HEC with CA as a matrix, which was further loaded with variable contents of ST nanoparticles. The samples were coded as HEC/CA as the matrix and HEC/CA/ST 5/10/20 as the composites, containing 5, 10, and 20 wt% ST, respectively. The original approach arises from the fact that the molecular interactions between the system counterparts can be modulated by the solvent action. Hence, two solvents were employed for the composite films’ preparation, namely H_2_O and H_2_O_2_. The oxidizing nature of the latter was expected to render complex reactions in the samples, which has the potential to advance the material’s optical and electrical properties.

## 2. Results

The attained composite materials are examined via several techniques to clarify the chemical structure and interactions by infrared spectroscopy; the homogeneity by scanning electron microscopy, thermal behavior by differential scanning calorimetry; and the optical features by UV-VIS-NIR spectroscopy, colorimetry, and refractometry, while the dielectric behavior was tested by broadband dielectric spectroscopy.

### 2.1. FTIR Investigations

**Figure 1:**
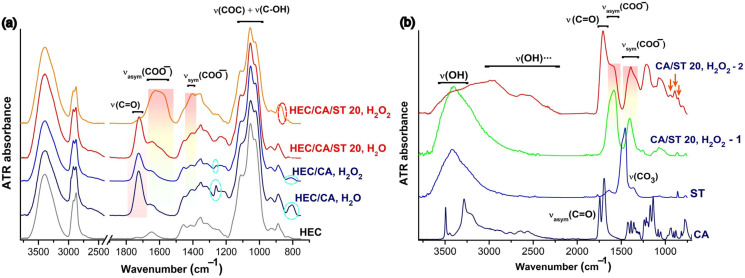
The infrared spectral analysis of HEC/CA/ST composites: (**a**) ATR-FTIR spectra of HEC-based films and (**b**) FTIR spectra of insoluble (green trace) and soluble (red trace) fractions of a CA/ST mixture in H_2_O_2_. The cyan dotted ellipses show the new bands at 1260 and 805 cm^−1^ for HEC/CA and HEC/CA/ST prepared in water. The red dotted ellipse shows the new shoulder at 865 cm^−1^ for the HEC/CA/ST prepared in H_2_O_2_. (**b**) The red arrows in (**b**) highlight the position of the triplet at 933, 883, and 868 cm^−1^, characteristic of peroxide ν(OO) vibrations (see text). The ν(OH···) assignment in (**b**) is for the H-bonded OH groups.

In the first place, the chemical structure of composites and the interactions between constituents were investigated by infrared spectroscopy. The ATR-FTIR spectra of the HEC-based films are presented in Figure 1a, in comparison to the native HEC. The major absorption bands given by the stretching vibrations of the alcoholic and polysaccharide backbone, observed around 3396 and 1054 cm^−1^, are almost identical in all HEC-based samples. For HEC/CA (prepared in either water or hydrogen peroxide) and HEC/CA/ST in water, new absorptions emerged at 1724, 1260, 1230, 865, and 805 cm^−1^. The bands at 1728, 1260, and 805 cm^−1^ can be assigned to ν(C=O), ν(COO), and δ(CO) of –O–C=O– acetal bridges that link HEC to CA [45,46].

**Scheme 1:**
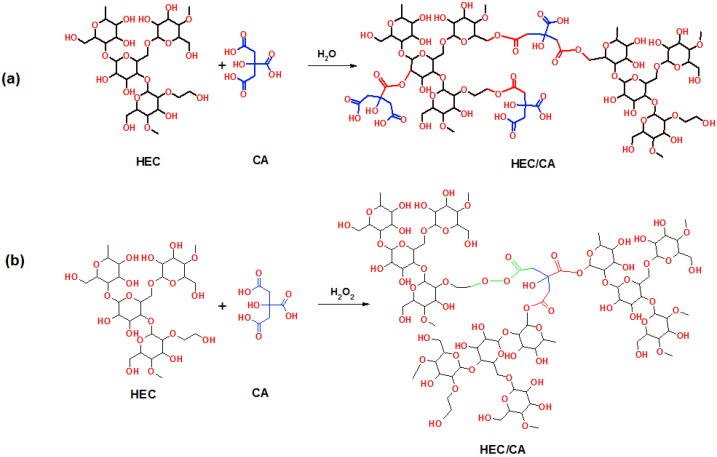
The proposed reactions leading to the structure of the HEC/CA matrix in (**a**) H_2_O and (**b**) H_2_O_2_. The peroxide bridge is highlighted in green color in (**b**).

For the HEC/CA sample prepared in H_2_O_2_ these vibrations are weaker, so one may conclude that using H_2_O_2_ as a solvent led to a smaller number of ester linkages. The hydrogen peroxide is a strong oxidizing agent and may oxidize some of HEC’s bonds and may react with CA, so finally some of the HEC-CA bridges may contain the peroxide O-O-group. The structure of the HEC/CA matrix can be described therefore by Scheme 1a,b, where HEC and CA are linked together by acetal or peroxide bridges.

Adding ST into the HEC/CA mixture resulted in two different kinds of samples. The FTIR spectrum of HEC/CA/ST films prepared in H_2_O is very similar with that of the HEC/CA matrix, showing the intact glucose band at 1054 cm^−1^ and the acetal ν(C=O) and ν(COO) vibrations at 1724 and 1234 cm^−1^, respectively. A new broad band around 1586 cm^−1^ appeared. The FTIR spectrum of HEC/CA/ST prepared in H_2_O_2_ is completely different from any of the above samples. It contains a broad and intense band between 1700 and 1500 cm^−1^ with two close maxima at 1623 and 1582 cm^−1^ and new peaks at 1405, 1372, 944, 883, and 868 cm^−1^. The first two sub-bands correspond to carboxylate ν(COO^−^) asymmetric stretching vibrations, in two different chemical species. The peak at 1402 cm^−1^ is the ν(COO^−^) symmetric vibration that correlates with ν_asym_(COO^−^) at 1582 cm^−1^ and suggests a bidentate bridging coordination of carboxylate—titanium complexes [46].

**Scheme 2:**
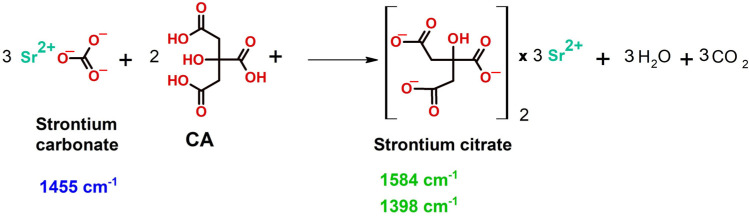
The proposed reaction of strontium carbonate with CA.

The bands at 1582 and 1402 cm^−1^ were identically reproduced in a side experiment, where CA has been mixed with ST in a H_2_O_2_ solution (Figure 1b). The insoluble and soluble fractions were analyzed individually, after removing the solvent. The blue trace in Figure 1b is the spectrum of the pure ST and mainly shows the CO_3_^−^ absorptions at 1455 and 861 cm^−1^ of the SrCO_3_ impurity and the weak Ti=O double bond at 938 cm^−1^ [47]. The green spectrum, denoted as CA/ST 20, H_2_O_2_—1, corresponds to the insoluble fraction of the CA/ST/H_2_O_2_ solution, which was a solid powder of a pale-yellow color. The red spectrum represents the soluble fraction (denoted CA/ST 20, H_2_O_2_—2), which is a yellow color. A comparison of the spectra between ST and the insoluble fraction indicates the lack of the 1455 cm^−1^ band and the presence of two intense bands at 1584 and 1398 cm^−1^, which are very specific to carboxylate asymmetric and symmetric stretching vibrations in the bidentate coordination [46]. It is assumed that the reaction between the SrCO_3_ impurity and CA occurred, according to Scheme 2, and strontium citrate is the main product:

The Ti=O band is shifted very little in either HEC/CA/ST—H_2_O_2_ (933 cm^−1^) or CA/ST 20, H_2_O_2_—2 (942 cm^−1^). The new band found at 883 cm^−1^ in HEC/CA/ST—H_2_O_2_, at 877 cm^−1^ in CA/ST 20, H_2_O_2_—1, and at 892 cm^−1^ in CA/ST 20, H_2_O_2_—2 is characteristic to the Ti-OOH vibration in η^1^ OOH^−^ hydroperoxide structures [48]. The band at 828 cm^−1^ in HEC/CA/ST—H_2_O_2_ and at 808 cm^−1^ in CA/ST 20, H_2_O_2_—2 is attributed to the Ti-O_2_ vibration in the triangular η^2^-OO^2−^ Ti–peroxide structure [48]. These absorptions are very characteristic of Ti–peroxo complexes and Ti–hydroperoxo complexes [48,49], formed by the interaction of ST with hydrogen peroxide. They are water-soluble and give the sample its yellow color [49].

The new vibrations at 1708, 1588, 1392, 1214, and 1075 cm^−1^ originate in the ν(C=O) in COOH, ν_asym_(COO^−^), ν_sym_(COO^−^), and ν(CO) vibrations, respectively. They suggest that the soluble fraction of CA/ST in H_2_O_2_ is composed of species where the citric acid is incompletely reacted at the acidic sites, because the ν(C=O) band at 1708 cm^−1^ is still present. At least one of the other two carboxyl functionalities interacts with ST in a deprotonated form. The reaction of Ti(IV) and Ti(III) with citric acid is well documented to lead to Titanium–citrate complexes of a general formula [Ti(CA*)(HCA*)_2_]^6−^ at pH 5.5 and [Ti(CA*)_3_]^8−^ at pH 7 [49]. Here, CA* is the fully deprotonated anionic form of CA, and HCA* is the normal, protonated structure. The formation of Ti(IV)−peroxo−citrate complexes with citrate as the chelating ligand has been reported by Kakihana [49,50] and Dakanali [51]. Generally, the precipitate is a mixture of mononuclear, dinuclear, or polynuclear complexes [49,50,51].

The very intense and broad ν(OH) band covers the 3700–2200 cm^−1^ region and indicates associations based on strong and short hydrogen bonds and oligomers and polymeric structures. Because of the similarities with the spectrum of carboxylic acids, we conclude that CA/ST 20, H_2_O_2_—2 is composed of assemblies based on -OOH●●●O-, -C(=O)OH●●●O-, and COO^−^●●●H^+^- bonds. Although all samples were very well dried before performing the IR analysis, the traces of hydrated/coordinated water appeared around 1630 cm^−1^ in every spectrum.

Based on the observations from above, the HEC/CA/ST–H_2_O_2_ film contains a complex mixture of Ti–peroxo and Ti–hydroperoxo complexes and Ti–citric acid complexes embedded within or in direct interaction with the HEC matrix. In contrast, HEC/CA/ST–H_2_O lacks the Ti–peroxide structures. The decomposition of hydrogen peroxide on the surface of SrTiO_3_ nanoparticles occurred similarly with the reported reactions for any other metal oxide [52,53]. The surface of the nanoparticles becomes enriched in hydroxyl, peroxide, and hydroperoxide groups. Hydroxylation would preferentially take place at the Sr^2+^ site [54], while the hydroperoxide Ti-OOH and the peroxide with the di-oxygen group bonded in the side-on η^2^ geometry are formed at the Ti^4+^ site [48,55]. Hydrogen peroxide decomposes into hydroxyl and hydroxyperoxyl radicals, with the formation of water and oxygen gas (Reactions (1)–(3)) [53]:

H_2_O_2_ → 2 OH● (1)

H_2_O_2_ + OH● → H_2_O + OOH● (2)

OH● + OOH● → H_2_O + O_2_ (gas)↑, (3)

The reactions between the oxygen ions of ST and OOH● and H_2_O_2_ and H_2_O produce SrTiO_3_-(OH) or, more generally, SrTiO_3_-(OH)*_n_* (Reactions (4)–(6)), which will act as anchors and reactive sites with the citric acid.

SrTiO_3_ + OOH● → SrTiO_3_-OH^−^ + O_2_^−^● (4)

2 H_2_O_2_ + SrTiO_3_ → H_2_O + SrTiO_3_-OH^−^ + OH^−^ (aq.) + O_2_ (gas) ↑ (5)

SrTiO_3_-OH^−^ + *n* H_2_O/H_2_O_2_ → SrTiO_3_-OH^−^ + *n* H_2_O (6)

From the interaction of hydroxylated SrTiO_3_ nanoparticles with citric acid, two complexes may result: either neutral ones, SrTiO_3_-(OH)*_n−m_* (COO-C_5_H_7_O_5_)*_m_* (Reaction (7)), or negative carboxylate complexes, SrTiO_3_-(OH_2_^+^)*_n_* (^−^COO-C_5_H_7_O_5_)*_x_* (Reaction (8)) [53]:

SrTiO_3_-(OH)*_n_* + *m* HCOO-C_5_H_7_O_5_ → SrTiO_3_-(OH)*_n-m_* (COO-C_5_H_7_O_5_)*_m_* + *m* H_2_O (7)

SrTiO_3_-(OH_2_^+^)*_n_* + *m* HCOO-C_5_H_7_O_5_ → SrTiO_3_-(OH)*_n_* (^−^COO-C_5_H_7_O_5_)*_x_* (8)

In the spectrum of HEC/CA/ST—H_2_O_2_ the presence of intense ν(COO^−^) vibrations altogether with ν(OH) (3396 cm^−1^) indicates that almost but not all carboxylic groups of CA have reacted with OH functionalities of ST through a bidentate coordination. A fraction of -OH groups from the surface of ST nanoparticles does not interact and contributes to the ν(OH) band.

As indicated above, a series of vibrations specific to Ti-O_2_ and Ti-OOH groups were observed for both HEC/CA/ST—H_2_O_2_ and CA/ST 20, H_2_O_2_—2. Their formation is attributed to the direct interaction between the Ti-O sites and the H_2_O_2_/H_2_O molecules, as described in Reactions (9) and (10) [55]. As judging from the magnitude of the corresponding bands, the fraction of Ti–hydroperoxide complexes is higher than that of ionic peroxo complexes, although the last ones are more stable.

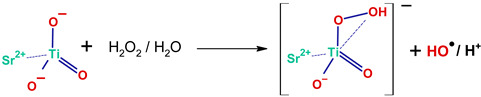
(9)
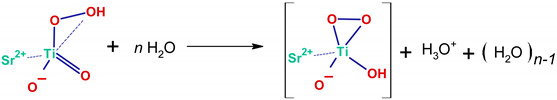
(10)


One may conclude that the combinations of ST, CA, and H_2_O_2_ have acted in synergy. They have redirected the reaction between HEC and CA to a reaction mainly between fillers and the solvent. The HEC matrix is less crosslinked by acetal bridges than the composite prepared in water and is filled with hydroperoxide titanium complexes with citrate (deprotonated and/or protonated) and with strontium citrate, in a lesser amount. Due to the high intensity of carboxylate vibrations, it is supposed that these metal complexes were preferentially deposited near the surface of the film.

### 2.2. Morphological Analysis

**Figure 2:**
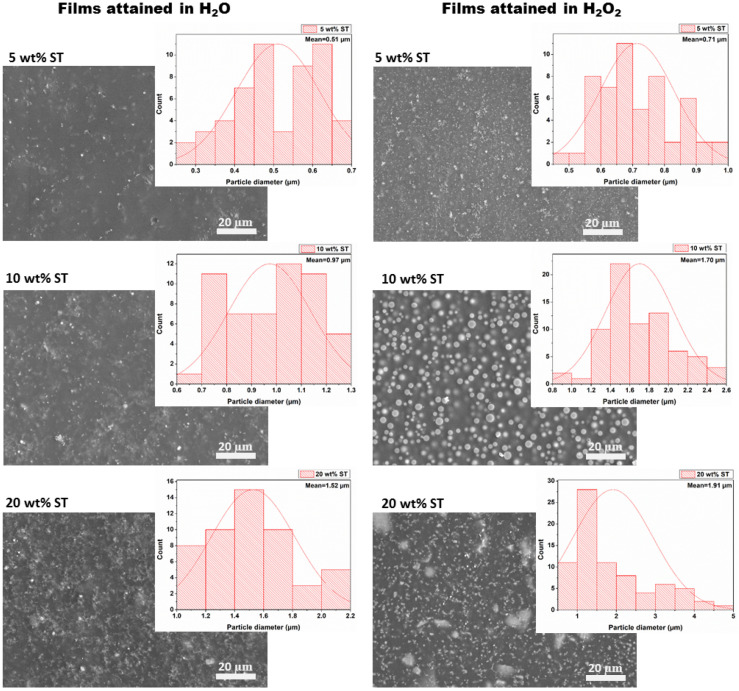
SEM images and the corresponding particle size distribution histograms of the HEC/CA/ST films containing several ST quantities (5–20 wt%), prepared in H_2_O and H_2_O_2_.

The morphology of the HEC/CA films containing distinct contents of ST (0–20 wt%) was examined to understand the effects produced by the loading and the nature of the used solvents. The SEM images illustrated in Figure 2 show that the two phases are properly mixed. The inorganic filler is observed as equally scattered white dots that are discernible at all loading levels. The size and the density of randomly distributed nanoparticles increase upon the progressive ST loading of the HEC/CA. The ceramic phase is well and uniformly dispersed in the polymer matrix. On the other hand, the solvent used affects the size of the nanoparticle agglomerates, which appear to be larger for the films attained in H_2_O_2_ comparatively with those processed in aqueous solutions (as observed from the particle size distribution histograms corresponding to each SEM image from Figure 2). In conjunction with the FTIR analysis, the nanoparticles seem to consist of aggregated Ti–peroxo complexes and Ti–hydroperoxo complexes, which have been excluded from the matrix during film drying.

### 2.3. DSC Analysis

**Figure 3:**
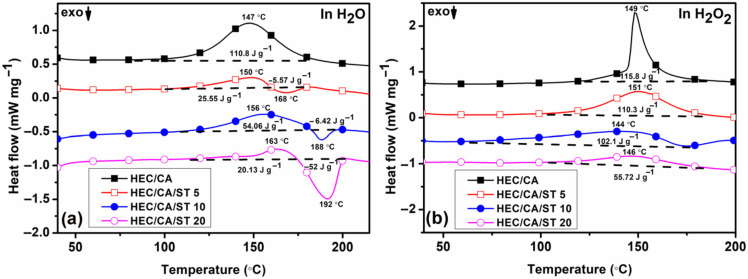
DSC curves of the studied films prepared in the two solvents: (**a**) H_2_O and (**b**) H_2_O_2_.

All DSC curves of the studied materials (Figure 3) show a broad endothermic profile associated with the solvent evaporation. The profile peak is located at 147 °C and 149 °C for the HEC/CA matrix in H_2_O and H_2_O_2_ media, respectively. From Figure 3, one may observe that the ST filler content and percentage increase lead to a significant lowering of the endothermic profile in both media (110.8 J g^−1^ vs. 20.13 J g^−1^ in H_2_O and 115.8 J g^−1^ vs. 55.72 J g^−1^ in H_2_O_2_) and its peak displacement toward generally higher temperature values. This is an indication that the filler presence and concentration significantly increase the nanocomposites’ hydrophobicity through blocking residual solvent retention. This aspect is crucial in most application domains, including specific electrical or electronic devices. Another interesting aspect is the apparition of an exothermic profile for the studied nanocomposites obtained only in H_2_O (Figure 3a). Unlike the solvent evaporation profile, this exothermic profile significantly increases both in intensity (–5.57 J g^−1^ vs. –52 J g^−1^) and temperature (168 °C vs. 192 °C) with ST percentage increases. This is due to the decomposition of the SrCO_3_, which inherently appears on the ST surface as result of the reaction with atmospheric carbon, as supported by the literature [56]. As already shown in the FTIR analysis, SrCO_3_ reacts with CA for the samples prepared in the H_2_O_2_ medium forming hydroperoxyde titanium complexes with citrate, which does not occur in the samples obtained in H_2_O. For this reason, this exothermic profile is discernible only for the films produced in water.

### 2.4. Optical Analysis

#### 2.4.1. Absorption Edge and Band Gap Energy

**Figure 4:**
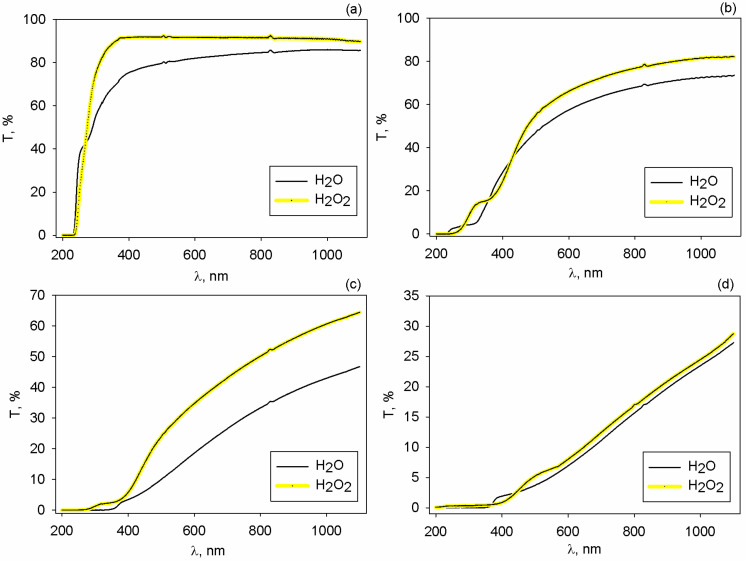
UV-VIS-NIR data for (**a**) HEC/CA, (**b**) HEC/CA/ST 5, (**c**) HEC/CA/ST 10, and (**d**) HEC/CA/ST 20 films prepared in the selected solvents.

The UV-VIS-NIR characteristics of the attained films are monitored in the wavelength domain of 200–1100 nm, as illustrated in Figure 4. Spectral features of the specimens are impacted not only by the amount of the inserted ceramic nanoparticles but also by the nature of the solvent. The analysis of the recorded data indicates lower values of the transparency for the films prepared in H_2_O regardless of the ST loading degree. By the gradual introduction of ceramic nanoparticles in the HEC/CA matrix, the level of transparency drops down, while the cut-off wavelength is moved towards higher values, namely 233 nm (0 wt% ST), 238 nm (5 wt%), 365 nm (10 wt%), and 374 nm (20 wt%) for films attained in H_2_O, while for those in H_2_O_2_, this parameter varies from 239 nm (0 wt% ST), 264 nm (5 wt%), 295 nm (10 wt%), and 406 nm (20 wt%).

Next, the absorption coefficient (α) is determined by applying the theory elaborated by Tauc [57] and Davis-Mott [58], which describes the variation in this optical parameter with the photon energy (hν) for amorphous materials. For the materials under investigation here, α was estimated according to Equation (11):

(11) $$\alpha =\frac{1}{t_{h}}ln\left(\frac{1}{T}\right),$$

where t_h_ is the specimen thickness, and T is the optical transmittance.

**Figure 5:**
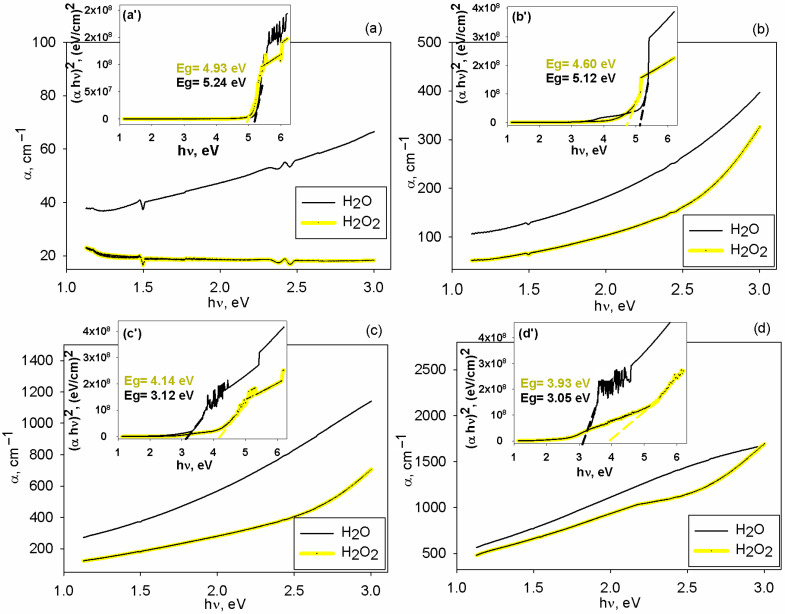
Plots of the absorption coefficient against the photon energy for (**a**) HEC/CA, (**b**) HEC/CA/ST 5, (**c**) HEC/CA/ST 10, and (**d**) HEC/CA/ST 20 films prepared in the selected solvents. The insets display the Tauc graphs for (**a’**) HEC/CA, (**b’**) HEC/CA/ST 5, (**c’**) HEC/CA/ST 10, and (**d’**) HEC/CA/ST 20 films.

The plots of α against hν for the samples are illustrated in Figure 5. One may see that the penetration level of the radiation in the HEC/CA-based materials is influenced by both the ST content and solvent type. The incorporation of ST nanoparticles with a larger polarizability in the matrix leads to a higher possibility of electron displacement upon the action of the light electrical field. This determines greater absorption coefficient values as the nanofiller amount is increased in the HEC/CA system. Also, the films obtained in H_2_O have higher values of α at all compositions comparatively with those in H_2_O_2_.

At energies higher than the optical gap, the α dependence on the hν is fitted by Tauc’s law [57], as indicated by Equation (12):

(12) $$\alpha E={C\left(E-E_{g}\right)}^{\beta },$$

where C is a constant and β is a parameter for electronic transitions, which might be equal to 1/2 or 2 for the direct and indirect transitions [59].

When plotting (α∙hν)^2^ versus hν, it is possible to evaluate the band gap energy (Eg), as seen in the inset images (a’)–(d’) from Figure 5. The literature reveals that ST has a direct band gap of 3.75 eV [60], while for the matrix components it was found that HEC HECHG has a direct Eg of 5.4 eV [61], and CA presents a direct Eg of 3.24 eV [62]. As often observed for most composites or blends, the addition of low-Eg compounds into materials (characterized by a wide forbidden gap) leads to an overall reduction effect on the magnitude of this parameter [63,64]. This is also the case for the studied specimens, where Eg values are decreasing as the amount of the ST nanoparticles increases in both H_2_O and H_2_O_2_ systems (see the insets from Figure 5). For the HEC/CA matrix and the composite with 5 wt% ST, the values of the direct band gap are higher for the films prepared in H_2_O comparatively with those achieved in H_2_O_2_. An opposite variation tendency is observed for the composites at higher ST loadings (10 wt% and 20 wt%).

#### 2.4.2. Colorimetry Analysis

A colorimetry examination of the specimens is further conducted to discern the effects of the ST loading and solvent type on the characteristics of the samples. The recorded values of the tristimulus parameters (X, Y, Z) for the prepared composites are included in Table 1.

For both systems obtained in the H_2_O and H_2_O_2_, the values of these parameters decrease upon the addition of the ST nanoparticles in regard to those attained for the HEC/CA matrix. Moreover, the specimens attained in H_2_O_2_ are characterized by higher values of X, Y, and Z comparatively with the films prepared from aqueous solutions. In order to comprehend how the chromatic features are evolving as a function of the composition and kind of solvent, the CIELAB coordinates (L*, a*, b*) were obtained, in agreement to Equations (13)–(15) [65]:

L* = 116 ∙ (Y/Yn)^1/3^ − 16, (13)

a* = 500 ∙ [(X/Xn)^1/3^ − (Y/Yn)^1/3^], (14)

b* = 200 ∙ [(Y/Yn)^1/3^ − (Z/Zn)^1/3^], (15)

where L* denotes the lightness parameter (0—black, 100—white), a* denotes the red/green parameter, and b* denotes the yellow/blue parameter, whilst Xn, Yn, and Zn represent the reference tristimulus information.

The results concerning the L*, a*, and b* parameters are listed in Table 1. One may observe greater lightness values as the loading degree of HEC/CA is lower, and also a higher L* is achieved for the specimens prepared in H_2_O_2_. The small and positive values of a* are indicative of a neutral hue of the matrix, while the presence of ST leads to the decrease in a* to negative values, meaning that the specimens tend to have green hues. This aspect is less accentuated for the samples processed in H_2_O. The positive values of b* indicate the yellowing shade of the samples as the ST amount increases in the HEC/CA. This can be observed even more so for the films prepared in H_2_O_2_. Further information can be acquired from the analysis of the yellowness index (YI), which is evaluated based on Equation (16) [66]:

YI = 142.86 ∙ (b*/L*),(16)

**Figure 6:**
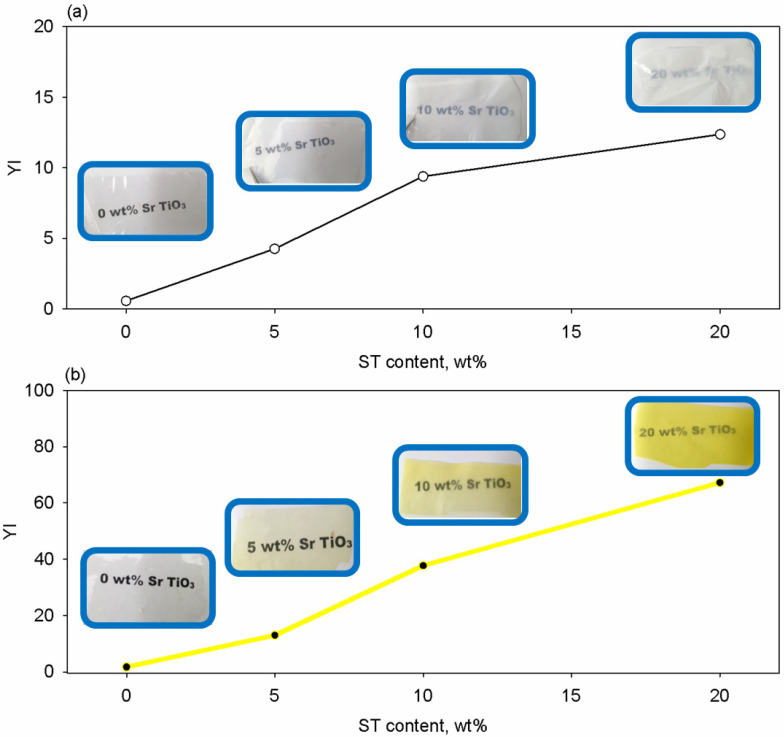
The changes in the yellowness index with the addition of variable contents of ST (0–20 wt%) for the HEC/CA films obtained in (**a**) H_2_O and (**b**) H_2_O_2_ and the corresponding pictures of the samples.

The modification of the YI in accordance with the sample’s composition and the solvent type is depicted in Figure 6. The values of the YI close to 0 for the HEC/CA films are indicative of a perfectly clear material, regardless of the solvent used. As the ST nanofillers are gradually introduced in the matrix, the magnitude of the YI increases as follows: from 4.26 (5 wt% ST) to 12.36 (20 wt% ST) for films obtained in H_2_O and from 1.71 (5 wt% ST) to 67.21 (20 wt% ST) for those in H_2_O_2_. It can be observed that the samples from aqueous solutions have smaller YI values (ranging from 0 to 12), emphasizing a whitening tendency. Moreover, at 20 wt% ST, the YI magnitude is increased by about 5.44 times for the layers processed in H_2_O_2_ in comparison to those in H_2_O. The YI parameter can be regarded as an indicator of the changes in the molecular interactions among the HEC, CA, and ST counterparts when the solvent nature differs. When the studied system is solved in an oxidizing medium, H_2_O_2_ interferes with the HEC and CA interactions and prevalently interacts with CA and ST (including SrCO_3_). In this case, SrCO_3_ contributes to the formation of complex compounds (i.e., Ti–peroxo complexes and Ti–hydroperoxo complexes) that differently absorb radiation (from violet/blue zone), thus shifting the sample’s color towards yellow. Conversely, when the same system is placed in water, the crosslinking of HEC by CA is not disturbed, while the ST nanoparticles generate light scattering across a wide range of wavelengths; hence, the films become whiter (SrCO_3_ here contributes to CO_2_ formation). All these factors determine the change in the aspect of the films from non-colored (HEC/CA samples) to a white (composites in H_2_O) or yellow (composites in H_2_O_2_) color, as seen in the sample’s pictures that are inserted in Figure 6.

#### 2.4.3. Refractive Index and Optical Dielectric Constant

The changes in the sample’s polarizability caused by the ST content and solvent type are monitored via refractivity experiments. The recorded data for the refractive index (n) at three wavelengths are presented in Table 2. The results show that the prepared films are characterized by normal dispersion features and that the incorporation of the ST nanoparticles produces the enhancement of the n values. The latter may be attributed to the formation of ceramic polar domains in the matrix that increase as the loading level of HEC/CA is higher. The oxidizing effects of the H_2_O_2_ on the system’s counterparts led to an additional increase in the n parameter.

The refractivity results are of great importance in energy-related applications. For instance, when designing flexible shielding covers for SCs, a good balance between a high transparency and the refractive index is prioritized. The composite containing 5 wt% ST in H_2_O_2_ appears to display an optimal trade-off between these optical properties, and this aspect is useful in practice to reduce the reflection losses at the interface with the ITO. For the classical glass cover, at a normal incidence, these are 1.73%, and for the mentioned sample the reflection losses drop down to 0.49%. On the other hand, the refractive index can be employed to determine the optical dielectric constant, as shown by Equation (17):

(17) $$\varepsilon_{\mathrm{opt}}=n^{2}-k^{2}$$

where k is the extinction coefficient, and ε_opt_ is the optical dielectric constant.

The data concerning the optical dielectric constant are included in Table 2. Similarly to the refractive index, the gradual addition of the ceramic nanofillers in HEC/CA produces the increase in ε_opt_ values due to the occurrence of ST domains of higher polarizability. This aspect is favorable for preparing dielectric layers suitable for EC applications.

### 2.5. Dielectric Behavior Analysis

**Figure 7:**
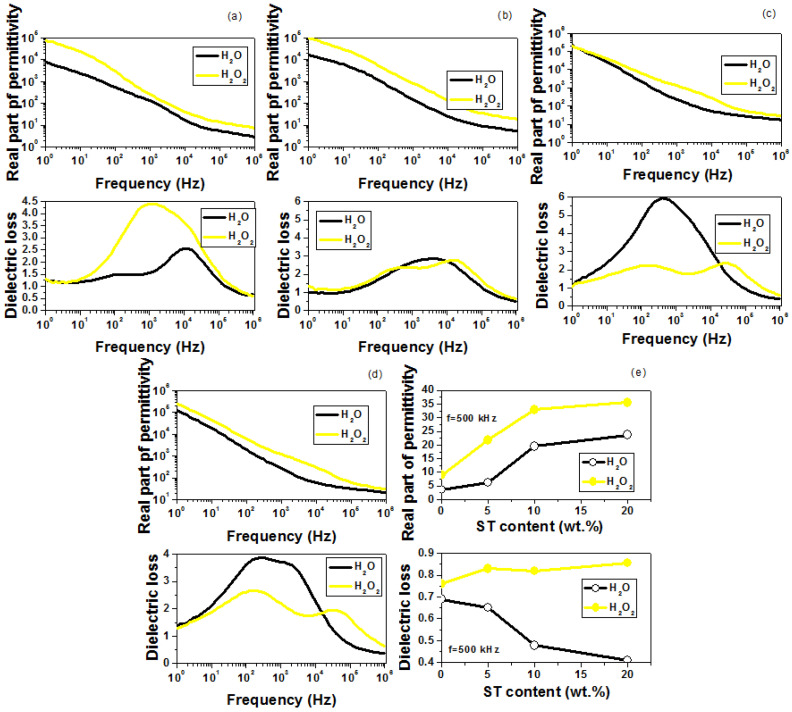
The frequency dependence of the real part of the permittivity and dielectric loss for (**a**) HEC/CA, (**b**) HEC/CA/ST 5, (**c**) HEC/CA/ST 10, and (**d**) HEC/CA/ST 20 films prepared in the selected solvents; (**e**) the compositional dependence of the real part of the permittivity and dielectric losses at f = 500 kHz.

The dielectric response of the samples was further investigated by dielectric spectroscopy. In Figure 7a–d the frequency dependence of the real part of the permittivity and dielectric loss for HEC/CA and HEC/CA/ST films in the selected solvents at room temperature is shown.

At low frequencies, all the samples show a Maxwell–Wagner-type relaxation attributed to the electrode effects and interfacial polarization [67]. The intrinsic dielectric properties of the material are revealed only for frequencies above 10 kHz. As a general trend, the permittivity values are larger for all the samples prepared in H_2_O_2_ in all frequency ranges. This behavior can be explained considering the FTIR observations: the samples prepared in H_2_O_2_ have a higher number of peroxide bridges, while in samples prepared in H_2_O the number of acetal bridges is prevalent. The last ones are not dipoles due to their high symmetry, while the peroxide bridges can act as dipoles due to their high reactivity and, implicitly, the ability to interact with substituents. Additionally, as can be seen from Figure 2, in the case of the samples prepared in H_2_O_2_, ST particles create larger clusters than in the case of the samples prepared in H_2_O. As shown in the literature [68], the clusters’ dimensions favor the permittivity increases. However, without any ST addition, the permittivity for HEC/CA is double (~7.5 at f = 500 kHz) for the film prepared in H_2_O_2_ compared to ~3.7 for that prepared in H_2_O. When ST is added, the effective permittivity increases (Figure 7e) from 7.5 for the HEC/CA film to about 35 for the 20 wt% ST (more than 4 times) in the case of the H_2_O_2_ solvent, while for the samples prepared in H_2_O it increases from 3.5 to 22 (~6 times). The values are in good agreement with the literature and our previous papers [38,42,69]. Furthermore, the dielectric losses at f = 500 kHz decrease from 0.7 to 0.4 for the samples prepared in water, while the samples prepared in H_2_O_2_ have almost the same losses ~0.8. However, the frequency dependence of losses (Figure 7a–d) show that for the composite samples the losses are larger at low frequencies (<1 kHz). Another observation is related to the two maxima at (i) ~10^2^ Hz and (ii) at 10^4^ Hz, which represent two relaxation phenomena with different relaxation times. In the case of the samples prepared in H_2_O, the two maxima are well delimitated in the pure film, while in the composites they overlap by shifting the maximum from 10^2^ to higher frequencies and the maximum from 10^4^ to lower frequencies, determining a maximum of losses for the 10 wt% ST. The behavior of samples prepared in H_2_O_2_ is the opposite: the pure HEC/CA presents an overlapping maximum that separates into two when increasing the ST concentration. Thus, the maximum losses will be in the neat HEC/CA film, while the composites display lower losses.

**Figure A1:**
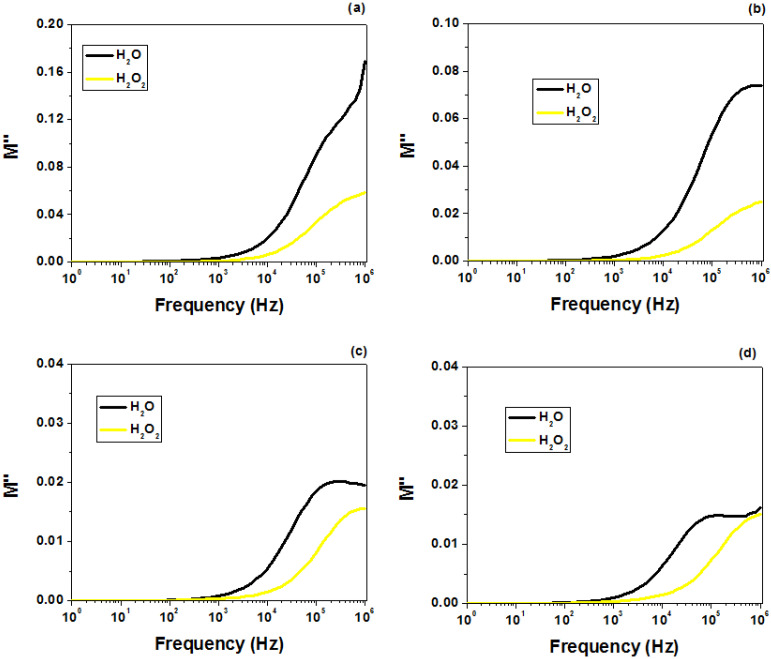
The frequency dependence of the imaginary part of the dielectric modulus for (**a**) HEC/CA, (**b**) HEC/CA/ST 5, (**c**) HEC/CA/ST 10, and (**d**) HEC/CA/ST 20 films prepared in the selected solvents.

**Figure A2:**
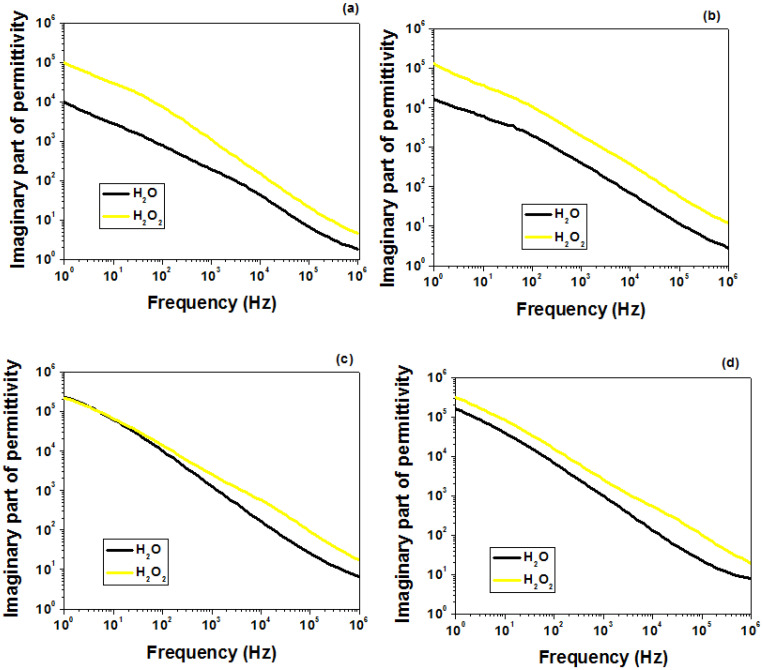
The frequency dependence of the imaginary part of the permittivity for (**a**) HEC/CA, (**b**) HEC/CA/ST 5, (**c**) HEC/CA/ST 10, and (**d**) HEC/CA/ST 20 films prepared in the selected solvents.

For a better understanding of the complex dielectric response, the dielectric modulus formalism was used in conjunction with a complex permittivity analysis. The variation in its imaginary component as a function of the frequency (Figure A1a–d and Figure A2a–d) provides information concerning the charge transport mechanisms, such as electrical transport and conductivity relaxation [70,71]. Conductivity relaxation can be identified by a peak in the M’’(f) spectra, with no corresponding peak in the ε’’(f) plot, whereas dielectric relaxation manifests maxima in both the imaginary component of the permittivity and the dielectric modulus. All the samples present a maximum between 10^5^ and 10^6^ in the M’’(f) spectra, whose magnitude decreases with the increasing ST content (Figure A1). In the case of the samples prepared in H_2_O, this maximum shift towards a lower frequency means an increase in the relaxation time of the polymer chain with increasing ST additions. For the samples prepared in H_2_O_2_, there seems to be a slight shift to higher frequencies, which means a reduction in the relaxation time and therefore an increase in the mobility of the polymer chain with the ST addition. This can be explained by considering the larger reactivity of samples prepared in H_2_O_2_. In the frequency dependence of the imaginary part of the permittivity, the relaxation mechanism is masked by electrode effects, and only a slight slope change is observed at frequencies between 1 and 10 kHz, and a decrease in the imaginary part of the permittivity in all frequency ranges is observed (Figure A2). From these data it can only be concluded that at a high frequency (~10^5^–10^6^ Hz) a relaxation of the conductivity occurs, without being able to extensively discuss the low frequency behavior.

**Figure 8:**
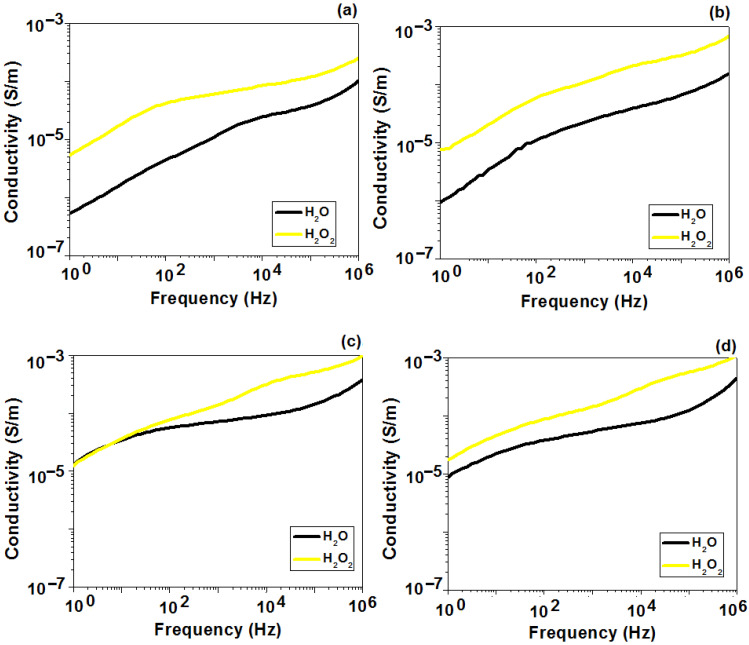
The frequency dependence of the effective conductivity at room temperature for (**a**) HEC/CA, (**b**) HEC/CA/ST 5, (**c**) HEC/CA/ST 10, and (**d**) HEC/CA/ST 20 films prepared in the selected solvents.

To elucidate between different contributions to the dielectric behavior, the frequency dependence of the conductivity was calculated and is represented in Figure 8. As already mentioned, the dielectric behavior at low frequencies is a combination of a heterogeneous conduction and the contribution of electronic polarization due to the polaron hopping mechanism. This behavior can be explained based on the polarization mechanisms both in the polymer and ST compounds. This mechanism is activated in this case by the solvent type and leads to an increase in the polarization at low frequencies, the dielectric constant, the losses, and the DC conductivity, as we have observed above. To understand the conduction mechanism and the type of polarons responsible for conduction in the two types of composites, we utilize the universal power law proposed by Jonscher [72].

## 3. Materials and Methods

### 3.1. Basic Materials

The polymer used in this article is hydroxyethyl cellulose (HEC) type NATROSOL 250 (Hercules Inc., Wilmington, DE, USA), which has molecular weight of 720 kDa (CAS number 9004-62-0).

Citric acid (CA) powder for analysis was acquired from Silal Trading SRL (Bucharest, Romania) and used as received (CAS number 5949-29-1).

Strontium titanate (ST) nanopowder (<100 nm particle size, 99% trace metal basis) was purchased from Sigma Aldrich (Saint Louis, MO, USA) and used as purchased (CAS number 12060-59-2).

Distilled water and hydrogen peroxide (3%, Vitalia SRL, Ploiesti, Romania) were used as solvents.

### 3.2. The Preparation of the Materials

**Scheme 3:**
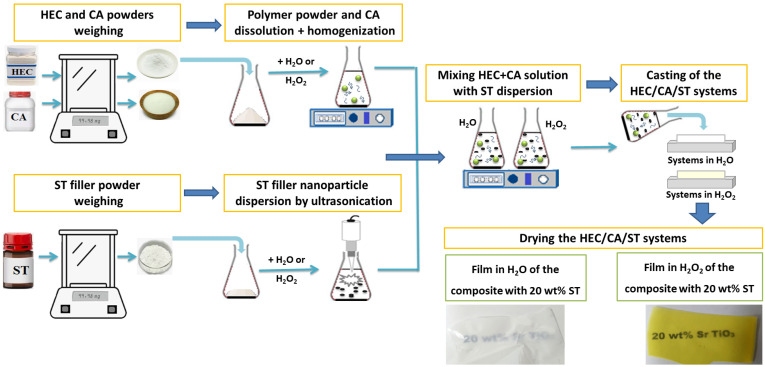
Basic preparation steps of the studied HEC/CA/ST materials.

A relatively simple procedure was employed for fabrication of the composite films, namely (a) the HEC polymer and CA powders were weighed, solubilized in 6 mL of solvent (H_2_O or H_2_O_2_), and homogenized by stirring for 6 h; (b) various ST nanopowder amounts were weighed (in 5, 10, and 20 wt% amounts) and dispersed in the chosen solvents by ultrasonication for 10 min on a VC-505 Ultrasonic Processor (Fisher Scientific, UK); (c) the HEC+CA solutions and the nanofiller dispersions were blended and stirred for 1 h; and (d) the systems were poured on glass slides and then dried in an oven at variable temperatures for 24 h. The basic preparation steps of the studied HEC/CA samples with variable ST content are illustrated in Scheme 3. The average thickness of the specimens was 38 µm.

### 3.3. Methods

Fourier transform infrared spectroscopy (FTIR) recordings of the films were carried out on a Bruker Vertex 70 spectrometer (Bruker Optics GMBh, Ettlingen, Germany) with a DTGS detector. The measurements were made in Attenuated Total Reflection mode, with the Golden Gate^TM^ equipment (Bruker Optics GMBh, Ettlingen, Germany) having a single-bounce diamond crystal, with 45° angle of incidence. The spectral region was 4000–600 cm^−1^. The spectra were recorded at 2 cm^−1^ resolution and as an average of 128 scans. The cyclic ether band at 1054 cm^−1^ was used as a reference in the spectral analysis.

Differential scanning calorimetry (DSC) measurements were conducted on a 200 F3 Maia apparatus (Netzsch–Gerätebau GmbH, Selb, Germany) in nitrogen atmosphere at a flow rate of 50 mL min^−1^. Around 6 mg of each sample was heated in aluminum crucibles with pierced and pressed lids at a heating rate of 10 °C min^−1^.

Scanning electron microscopy examination of the films was conducted on Verios G4 UC instrument (Thermo Scientific, Brno, Czech Republic). A statistical image analysis using ImageJ software (version 1.52a, Java 1.8.0_112) was conducted on the SEM images, providing particle size distribution histograms for each sample examined. The average aggregate diameters were determined by assessing 50–60 measurements per sample.

The transmittance in UV-VIS domain of the films (2 cm × 2 cm) was measured on a SPECORD 210 PLUS apparatus (Analytik Jena GmbH, Jena, Germany).

Colorimetry analyses of the films were undertaken on a CL-70F instrument (Konika Minolta, Tokyo, Japan).

Refractometry analysis was performed (at 25 °C) on a refractometer (Anton Paar GmbH, Ashland, VA, USA) equipped with three wavelength filters.

Dielectric behavior of the films was examined on Solartron 1260 system (Solartron Analytical, Hampshire, Farnborough, UK) at room temperature at frequency varying between 1 Hz and 1 MHz.

## 4. Conclusions

This work investigated a new pathway for tailoring the properties of biodegradable polymers, not only via composition but also via the solvent-driven interactions. The composite specimens were attained by the ST loading (5–20 wt%) of a matrix composed of a cellulose ether/citric acid mixture in H_2_O or H_2_O_2_. Depending on the solvent nature, CA can react mainly with the polymer or with the inorganic nanofiller, resulting in considerable differences in the overall physical properties of the composites. The FTIR results emphasized that in H_2_O, HEC is crosslinked by CA via acetal bridges, while in H_2_O_2_, the main reaction is between the solvent, CA, and ST, because they are much more reactive than HEC. So, in an oxidizing environment the polymer was less crosslinked by the ester or peroxo bridges than the composite prepared in H_2_O and was filled with peroxo–citrate titanium complexes and with strontium citrate. The morphological features of the matrix were changed by both the ST loading and by the solvent, which in the case of H_2_O_2_ favored the occurrence of larger nanofiller agglomerates. DSC data reveal that the presence of the ST filler determines a significant lowering of the endothermic profile of the samples prepared in both solvents, indicating an enhancement of the hydrophobicity through blocking the residual solvent retention. Optical analyses indicated that H_2_O_2_ led to a greater transparency, greater yellowness, and enhanced refractive index (implicitly optical dielectric constant). Furthermore, the dielectric characteristics as a function of frequency indicate an increase in the permittivity (about 4–6 times for 20% ST filler) accompanied by a reduction in the frequency dependence of dielectric losses with ST additions. Also, the films prepared in H_2_O_2_ presented a higher permittivity and smaller losses for all samples investigated. These data provide new possibilities to adjust the optical and dielectric behavior of eco-friendly materials for designing components needed in energy-related devices, such as shielding covers for solar cells or dielectric layers for electrostatic capacitors.

## Figures and Tables

**Table 1:** The recorded tristimulus parameters and the calculated L*—lightness, a*—red/green parameter, and b*—yellow/blue parameter.

System	ST, wt%	X	Y	Z	L*	a*	b*
HEC/CA/ST films prepared in H_2_O	0	384.4128	399.9580	429.7695	93.0786	0.2584	0.3728
	5	370.4469	386.3166	399.3975	91.8241	−0.1016	2.7398
	10	329.0398	343.4064	336.4649	87.6743	−0.2152	5.7599
	20	249.2407	259.9917	247.3358	78.4905	−0.1275	6.7915
HEC/CA/ST films prepared in H_2_O_2_	0	414.9604	431.9948	458.6668	95.9166	0.1706	1.1499
	5	396.2388	420.8070	396.2108	94.9420	−2.9978	8.6038
	10	357.8094	393.8825	279.7866	92.5235	−8.4612	244371
	20	311.7227	352.9012	173.3759	88.6211	−12.2736	41.6947

**Table 2:** The refractive index and the optical dielectric constant.

System	ST, wt%	n			ε_opt_		
		486 nm	589 nm	656 nm	486 nm	589 nm	656 nm
HEC/CA/ST films prepared in H20	0	1.5049	1.4977	1.4968	2.2647	2.2431	2.2404
	5	1.5152	1.5063	1.5022	2.2958	2.2689	2.2566
	10	1.5190	1.5177	1.5118	2.3073	2.3034	2.2855
	20	1.5646	1.5471	1.5334	2.4479	2.3935	2.3513
HEC/CA/ST films prepared in H202	0	1.5090	1.4993	1.4981	2.2771	2.2479	2.2443
	5	1.5229	1.5189	1.5132	2.3192	2.3071	2.2898
	10	1.5843	1.5705	1.5606	2.5100	2.4665	2.4355
	20	1.5914	1.5811	1.5763	2.5325	2.4999	2.4847

## Data Availability

The original contributions presented in this study are included in the article. Further inquiries can be directed to the corresponding author.

## Appendix A

The variation in the imaginary component of the dielectric modulus as a function of frequency is given in Figure A1.

The variation in the imaginary component of the permittivity as a function of frequency is given in Figure A2.
